# Association of sleep-wake rhythm and sleep quality with endothelial function in young adults

**DOI:** 10.5935/1984-0063.20220050

**Published:** 2022

**Authors:** Honoka Nakashima, Akiko Noda, Anna Tamura, Michiaki Nagai, Masato Okuda, Takahiro Okumura, Fumihiko Yasuma, Toyoaki Murohara

**Affiliations:** 1 Chubu University Graduate School of Life and Health Sciences, Department of Biomedical Sciences - Kasugai - Aichi - Japan; 2 Chubu University Collage of Life and Health Sciences, Department of Biomedical Sciences - Kasugai - Aichi - Japan; 3 Hiroshima City Asa Hospital, Hiroshima, Department of Cardiology - Hiroshima - Japan; 4 Nagoya University Graduate School of Medicine, Department of Cardiology - Nagoya - Aichi - Japan

**Keywords:** Circadian Rhythm, Young Adult, Actigraphy

## Abstract

**Objective:**

The environment in modern society could disturb the sleep-wake rhythm. We aimed to study the association of sleep-wake rhythm with endothelial function and sleep quality.

**Material and Methods:**

Thirty-one healthy university students (mean age: 20.4±1.8 years) were enrolled. The endothelial function was evaluated with the percent endothelium-dependent flow-mediated dilation of the brachial artery [%FMD: (maximum diameter - baseline diameter)/baseline diameter x 100] using the high-resolution ultrasonography. We also measured the total sleep time (TST), sleep effciency, and the standard deviation (SD) of sleep timing (midpoint between bedtime and wake-up time) using the actigraphy. The irregular sleep-wake rhythm was defined as having the shift of bedtime or wake-up time for two hours or longer.

**Results:**

The %FMD and sleep efficiency were significantly lower in the irregular group than regular group (%FMD: 6.1±2.4 vs. 10.9±2.3, *p*<0.001, sleep effciency: 92.2±5.8 vs. 95.9±2.8%, *p*=0.027), whereas there was no significant difference in %FMD between the two groups of TST <6 hours and TST ≥6 hours. The %FMD was significantly correlated with SD of sleep timing (*r*=-0.481, *p*=0.006). Multiple regression analyses, including age, sex, TST, sleep effciency, and SD of sleep timing revealed that the SD of sleep timing was a significant factor associated with %FMD (*ß*=-0.454, *p*=0.017).

**Conclusion:**

Our findings suggest that the irregular sleep-wake rhythm and poor sleep quality could have adverse effects on endothelial function in young adults.

## INTRODUCTION

The environment in modern society could often disturb the sleep-wake rhythm^1^. Many of the university students have irregular schedules of classes^2^, and part-time works^3^. Moreover, they use the internet and social networking service on 24-hour basis^4^,^5^, with frequent shiftings of the bedtime and wake-up time. Accumulated sleep debt reduces their quality of sleep^6^, which has been identified as a major cause of fatigue, inattentiveness, anxiety^7^, and depression^8^. We recently showed that an irregular sleep-wake rhythm and a short sleep duration played a negative role in the brain activity of the university students using near infrared spectroscopy^9^. However, the association between the sleep-wake rhythm and endothelial function has not been clarified in young adults.

Endothelial dysfunction with impaired nitric oxide (NO) production is an early feature of atherosclerosis and cardiovascular diseases in human^10^. The NO is a product of endothelial cells that regulates the vascular tone and it plays a pivotal role for maintaining the homeostasis in hemodynamics. Since significant day-to-day variations in sleep duration and timing are assumed to be associated with the future risk of atherosclerosis^11^,^12^, lifestyle interventions for young adults could prevent occurrences of cardiovascular diseases in the long run.

Accordingly, we investigated the effects of sleep-wake rhythm and sleep quality on endothelial function in the university students.

## MATERIAL AND METHODS

### Subjects

Thirty-one healthy university students (mean age: 20.4±1.8 years) were enrolled in this study. None of these subjects had any history of neurological disorder, substance abuse, head injury or major physical illness. They were free from smoking and did not use psychotropic medication. This study was approved by the ethics committee of Chubu University (Number 270098). After explaining the nature of the study and procedures involved, we obtained written informed consents from all participants.

### Actigraphy

Actigraphy for monitoring the activities and scoring the sleep-wake rhythm (Ambulatory Monitoring Inc., New York, NY, USA) was performed for 7 consecutive days. The actigraph was worn around the wrist of their non-dominant side to store the data in 1-min increments. Bedtime and wake-up time were derived from the sleep diary, with which the analysis interval of actigraphy was ascertained^13^. We used the algorithm supplied by the ActionW-2 clinical sleep analysis software package for Windows (Ambulatory Monitoring Inc., New York, NY, USA) to score the sleep/wakefulness according to the Cole-Kripke formula^14^. We evaluated total sleep time (TST), sleep efficiency (calculated as TST/time spent in bed x 100), bedtime, wake-up time, sleep timing (midpoint between bedtime and wake-up time), and standard deviation (SD) of sleep timing^15^. The irregular sleep-wake rhythm was defined as having the shift of bedtime or wake-up time for two hours longer according to the International Classification of Sleep Disorders, 3^rd^ edition^16^. We classified the participants into two groups of irregular group (n=16) and regular group (n=15) in sleep-wake rhythm.

### Brachial-ankle pulse wave velocity (baPWV)

The baPWV was measured using a plethysmograph (BP-203RPEIII, Omron Colin, Tokyo, Japan)^17^, and systolic blood pressure (SBP), diastolic blood pressure (DBP), and heart rate (HR), electrocardiogram (ECG), and heart sounds were simultaneously recorded with this instrument.

### Endothelium-dependent flow-mediated dilation (FMD)

The FMD of the brachial artery was assessed using the high-resolution ultrasonography equipped with a 12MHz linear array transducer (Prosound α7, Hitachi Aloka Medical, Tokyo, Japan)^18^,^19^. The diastolic time was identified continuously with three-lead ECG. A sphygmomanometric cuff on the right distal forearm was utilized for creating a flow stimulus to the brachial artery. After the diameter of brachial artery was measured at baseline at rest, the cuff was inflated with 50mmHg above the SBP to occlude the brachial artery for 5 minutes and subsequently deflated for 3 minutes to restore the flow. Endothelial function was assessed as a change in the diameter of brachial artery from baseline to peak expansion after cuff release. The %FMD was defined as: (maximum diameter - baseline diameter)/baseline diameter x 100.

### Statistical analysis

All data are expressed as mean ± SD. We compared the data on SBP, DBP, HR, baPWV, %FMD, TST, sleep efficiency, bedtime, wake-up time, sleep timing, and SD of sleep timing between the groups (irregular sleep-wake rhythm vs. regular sleep-wake rhythm, and TST <6 hours vs. TST ≥6 hours^20^) using the non-paired *t*-test. The Pearson’s correlation analyses were performed to evaluate the relationships between the %FMD and sleep parameters. In addition, TST, sleep efficiency, and SD of sleep timing were included in multiple regression analyses to determine the independent parameters correlated with %FMD or baPWV. A probability value less than 0.05 was considered statistically significant. All statistical analyses were performed using the SPSS Statistics version 25.0 (IBM Corporation, Armonk, New York, USA).

## RESULTS

**Figure 1:**
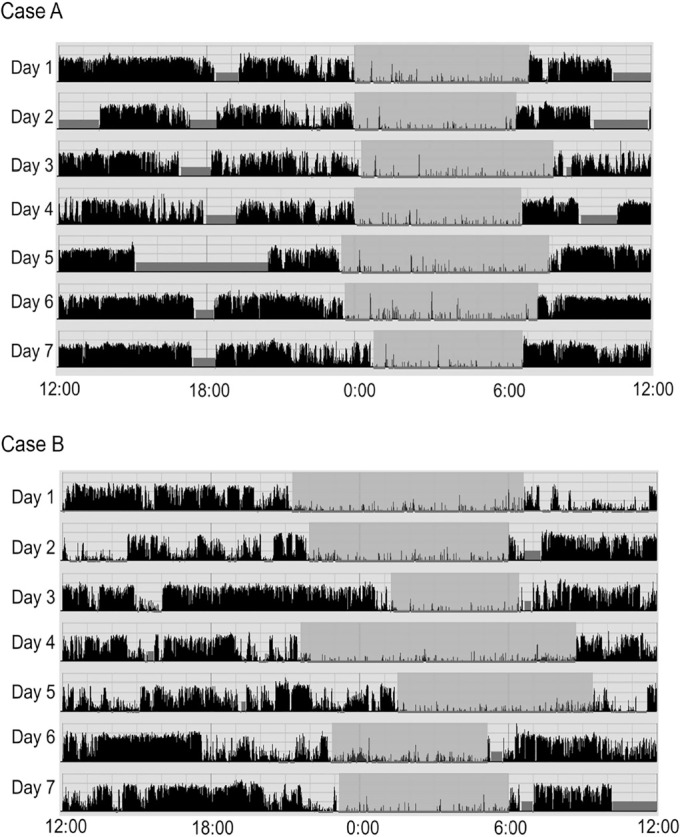
Actigram of two representative cases of regular sleep-wake rhythm (case A) and irregular sleep-wake rhythm (case B).

Notes: The horizontal axis represents the clock over 24 hours (12 p.m. to 12 p.m.). The vertical axis represents the amount of activity recorded by the actigraph, with the black bars indicating the movement in 1 min. The section of  indicates the period when the participants were in bed and the section of  indicates the period when the participants apparently removed the actigraphy instrument.

Table 1 shows the characteristics, vascular function and sleep parameters of the participants. Figure 1 shows two representative actigrams of regular sleep-wake rhythm (Case A) and irregular sleep-wake rhythm (Case B).

**Table 1:** Demographics and vascular/sleep parameters.

Demographics	
Gender (male/female)	9/22
Age (years)	20.4 ± 1.8
Height (cm)	161.0 ± 7.4
Weight (kg)	54.2 ± 10.2
BMI (kg/m^2^)	20.9 ± 2.7
**Vascular parameters**	
SBP (mmHg)	112.2 ± 11.5
DBP (mmHg)	61.9 ± 5.2
HR (bpm)	67.3 ± 9.2
baPWV (cm/s)	973.5 ± 198.3
%FMD	8.4 ± 3.4
**Sleep parameters**	
TST (min)	366.3 ± 58.5
Sleep efficiency (%)	94.2 ± 4.9
Bedtime	1:13 ± 0:58
Wake-up time	7:52 ± 0:52
Sleep timing	4:39 ± 0:55
SD of sleep timing (min)	69.7 ± 42.5

Notes: Data are expressed as mean ± standard deviation. BMI = Body mass index; SBP = Systolic blood pressure; DBP = Diastolic blood pressure; HR = Heart rate; baPWV = Brachial-ankle pulse wave velocity; FMD = Flow-mediated dilation; TST = Total sleep time; SD = Standard deviation.

The SD of sleep timing was significantly greater in the group of TST <6 hours than group of TST ≥6 hours (86.2±49.2 vs. 56.0±28.5min, *p*=0.041). No significant difference was observed in %FMD between the groups (Table 2).

**Table 2:** Comparison of demographics and vascular/sleep parameters by total sleep time.

	TST <6 hours (n=14)	TST ≥6 hours (n=17)	p-value
**Demographics**			
Age (years)	20.1 ± 1.7	20.6 ± 1.8	0.494
BMI (kg/m^2^)	20.6 ± 1.9	21.2 ± 3.3	0.525
**Vascular parameters**			
SBP (mmHg)	108.8 ± 7.6	115.0 ± 13.8	0.143
DBP (mmHg)	60.9 ± 5.4	62.7 ± 6.6	0.440
HR (bpm)	65.9 ± 10.1	68.5 ± 8.5	0.452
baPWV (cm/s)	986.3 ± 125.7	963.1 ± 245.9	0.751
%FMD	8.1 ± 3.5	8.7 ± 3.3	0.645
**Sleep parameters**			
Sleep efficiency (%)	94.1 ± 4.9	94.3 ± 4.7	0.932
Bedtime	1:24 ± 0:43	1:04 ± 1:05	0.662
Wake-up time	7:47 ± 1:02	7:56 ± 0:49	0.321
Sleep timing	4:52 ± 0:57	4:28 ± 0:52	0.221
SD of sleep timing (min)	86.2 ± 49.2	56.0 ± 28.5	0.041

Notes: Data are expressed as mean ± standard deviation. TST = Total sleep time; BMI = Body mass index; SBP = Systolic blood pressure; DBP = Diastolic blood pressure; HR = Heart rate; baPWV = Brachial-ankle pulse wave velocity; FMD = Flow-mediated dilation; SD = Standard deviation.

The %FMD and sleep efficiency were significantly lower in the irregular group than regular group in sleep-wake rhythm (%FMD: 6.1±2.4 vs. 10.9±2.3, *p*<0.001, sleep efficiency: 92.2±5.8 vs. 95.9±2.8%, *p*=0.027). Moreover, the sleep timing and SD of sleep timing were significantly greater in the irregular group than regular group (sleep timing: 4:58±0:52 vs. 4:18±0:52, *p*=0.042, SD of sleep timing: 94.3±29.6 vs. 43.3±36.0min, *p*<0.001) (Table 3).

**Table 3:** Comparison of demographics and vascular/sleep parameters by sleep-wake rhythm.

	Irregular group (n=16)	Regular group (n=15)	p-value
**Demographics**			
Age (years)	20.9 ± 1.7	20.0 ± 1.7	0.113
BMI (kg/m^2^)	20.3 ± 1.9	21.6 ± 3.3	0.196
**Vascular parameters**			
SBP (mmHg)	108.9 ± 8.3	115.7 ± 13.9	0.110
DBP (mmHg)	61.3 ± 6.6	62.5 ± 5.5	0.604
HR (bpm)	67.6 ± 9.0	67.0 ± 9.6	0.853
baPWV (cm/s)	918.3 ± 249.2	1032.5 ± 101.6	0.110
%FMD	6.1 ± 2.4	10.9 ± 2.3	< 0.001
**Sleep parameters**			
TST (min)	353.8 ± 70.2	379.7 ± 51.4	0.253
Sleep efficiency (%)	92.2 ± 5.8	95.9 ± 2.8	0.027
Bedtime	1:28 ± 0:54	0:56 ± 0:58	0.132
Wake-up time	8:08 ± 0:58	7:35 ± 0:47	0.105
Sleep timing	4:58 ± 0:52	4:18 ± 0:52	0.042
SD of sleep timing (min)	94.3 ± 29.6	43.3 ± 36.0	< 0.001

Notes: Data are expressed as mean ± standard deviation. BMI = Body mass index; SBP = Systolic blood pressure; DBP = Diastolic blood pressure; HR = Heart rate; baPWV = Brachial-ankle pulse wave velocity; FMD = Flow-mediated dilation; TST = Total sleep time; SD = Standard deviation.

Significant correlation was observed between %FMD and the SD of sleep timing (*r*=-0.481, *p*=0.006) in the simple correlation analysis. In the multiple regression analysis, the SD of sleep timing was a significant factor associated with %FMD (*β*=-0.454, *p*=0.017) (Table 4).

**Table 4:** Relationships among vascular and sleep parameters.

	Simple correlation analysis		Multiple regression analysis	
	**r**	**p-value**	**β**	**p-value**
**%FMD**				
TST	0.186	0.315	0.001	0.996
Sleep efficiency	0.225	0.224	0.138	0.434
SD of sleep timing	-0.481	0.006	-0.454	0.017
**baPWV**				
TST	-0.227	0.219	-0.287	0.165
Sleep efficiency	0.061	0.743	0.119	0.542
SD of sleep timing	-0.015	0.937	-0.086	0.665

Notes: FMD = Flow-mediated dilation; TST = Total sleep time; SD = Standard deviation; baPWV = Brachial-ankle pulse wave velocity.

## DISCUSSION

We found that an irregular sleep-wake rhythm was adversely affecting endothelial function. Moreover, sleep efficiency was significantly lower in young adults with irregular sleep-wake rhythm than those with regular sleep-wake rhythm. Our findings suggest that irregular sleep-wake rhythm may be associated with reduced endothelial function and poor sleep quality.

Many of the Japanese university students devote their free time to a part-time job, use social networking and other site on a 24-hour basis^4^-^6^, or engage in-out-of class activities. Thus, irregular sleep schedule may be a result of these lifestyle choices. Irregular sleep-wake rhythm might disturb behavioral rhythms in regards to timing/amount of eating, which can pose even higher cardiovascular risks to irregular sleepers of nocturnal food intake and breakfast skipping^21^-^23^ or reduced physical activity^24^,^25^.

The irregular sleep-wake rhythm of a long-term intermittent night shift worker was a factor of impaired endothelial function^26^,^27^, with most shift workers exhibiting a significant increase in arterial stiffness. Moreover, in a multicenter, cross-sectional, population-based study, the late timing of sleep and irregular sleep-wake rhythm were associated with hypertension, suggesting that interventions to adjust the timing of sleep and obtain a regular sleep-wake rhythm may be important targets for cardiovascular health^28^. These findings may explain the relationship between the irregular sleep-wake rhythm and endothelial dysfunction in young adults.

In a study of BMAL1-knockout and clock mutant mice, aberrant circadian rhythms impaired the endothelial function presumably via a decrease in NO production^29^. Other studies on myocardial ischemia-induced mice demonstrated that recovery from ischemia was delayed by the aberrant circadian rhythms^30^, and the tolerance for myocardial infarction was improved after modifying of circadian rhythms^31^. Disturbed diurnal rhythm was found to alter gene expression, which affected the cardiovascular system adversely^32^. Hence, the disruption of sleep-wake rhythms may precipitate endothelial dysfunction, which would lead to future occurrence of cardiovascular diseases.

An irregular sleep-wake rhythm was associated with reduced endothelial function and lower sleep efficiency. Hypothalamic-pituitary-adrenal axis dysregulation was associated with the sympathovagal imbalance in sleep deprivation^33^,^34^. A disruption of circadian rhythm and poor sleep quality have also been found to influence the rhythms of the autonomic nervous system^35^,^36^, which directly govern normal cardiac function. Thus, the sympathovagal imbalance may be involved in endothelial dysfunction in young adults. Lowered sleep efficiency stimulated the secretions of Interleukin-6 and C-reactive protein, which were known to impair the endothelial function^37^,^38^. Moreover, long-term inflammation and excessive reactive oxygen species promote oxidative stress, which could lead to the endothelial dysfunction^39^. A previous study assessed FMD in adults free from routine work and showed that poor sleep quality was associated with the endothelial dysfunction^40^. Therefore, a regular sleep-wake rhythm may play an important role in maintaining the sleep quality, sympathovagal balance, and cardiovascular health in even young adults.

In this study, we showed that the TST was not a significant factor affecting the FMD. In a cross-sectional study of 684 subjects (32% male, 68% female) aged 37 to 60 years, there was a significant relationship only seen between the FMD and component 1 (sleep quality) of Pittsburgh sleep quality index, but not with sleep duration^41^. Short sleep duration was not associated with a dysfunction of circulating endothelial progenitor cells in thirty-seven healthy adults (age range: 43-65 years)^42^. In addition, the short sleep duration was associated with in reduced FMD in healthy adults, but not with the reduced FMD after adjusting for sex, age, body mass index, smoking, and other complications^43^. Moreover, the TST in the polysomnographic study was not associated with FMD^40^. Our present results were consistent with the relationship between sleep duration and endothelial function in the previous studies.

The present study has methodological limitations. The study population was relatively small and the sample size was not sufficient to examine the gender differences. Especially, hormones and the menstrual cycle might have biased the results^44^. In the future, prospective or interventional trials with larger sample sizes are required to elucidate the causal relationship between sleep parameters and endothelial function.

## CONCLUSION

An irregular sleep-wake rhythm was associated with the reduced endothelial function and lower sleep efficiency in young adults. Thus, a lifestyle promoting regular sleep-wake rhythm is important for benefits such as better sleep quality and cardiovascular health.
